# A novel and innovative cancer classification framework through a consecutive utilization of hybrid feature selection

**DOI:** 10.1186/s12859-023-05605-5

**Published:** 2023-12-15

**Authors:** Rajul Mahto, Saboor Uddin Ahmed, Rizwan ur Rahman, Rabia Musheer Aziz, Priyanka Roy, Saurav Mallik, Aimin Li, Mohd Asif Shah

**Affiliations:** 1 School of Computing Science and Engineering, VIT Bhopal University, Kothrikalan, Sehore, Madhya Pradesh 46611 India; 2 School of Advanced Sciences and Language, VIT Bhopal University, Kothrikalan, Sehore, Madhya Pradesh 46611 India; 3 Molecular and Integrative Physiological Sciences, Department of Environmental Health, Harvard T. H. Chan School of Public Health, Boston, MA 02115 USA; 4 Department of Pharmacology and Toxicology, University of Arizona, Tucson, AZ 85721 USA; 5 Center for Precision Health, School of Biomedical Informatics, The University of Texas Health Science Center at Houston, Houston, TX 77030 USA; 6 School of Computer Science and Engineering, Xi’an University of Technology, Shaanxi, 710048 China; 7 Department of Economics, Kebri Dehar University, Kebri Dehar, 250 Somali Ethiopia; 8 Division of Research and Development, Lovely Professional University, Phagwara, Punjab 144001 India; 9 Centre for Research Impact & Outcome, Chitkara University, Rajpura, Punjab 140401 India

**Keywords:** Deep learning (DL), Cuckoo search algorithm (CSA), Spider monkey optimization (SM), Minimum redundancy maximum relevance (mRMR), Cancer classification

## Abstract

Cancer prediction in the early stage is a topic of major interest in medicine since it allows accurate and efficient actions for successful medical treatments of cancer. Mostly cancer datasets contain various gene expression levels as features with less samples, so firstly there is a need to eliminate similar features to permit faster convergence rate of classification algorithms. These features (genes) enable us to identify cancer disease, choose the best prescription to prevent cancer and discover deviations amid different techniques. To resolve this problem, we proposed a hybrid novel technique CSSMO-based gene selection for cancer classification. First, we made alteration of the fitness of spider monkey optimization (SMO) with cuckoo search algorithm (CSA) algorithm viz., CSSMO for feature selection, which helps to combine the benefit of both metaheuristic algorithms to discover a subset of genes which helps to predict a cancer disease in early stage. Further, to enhance the accuracy of the CSSMO algorithm, we choose a cleaning process, minimum redundancy maximum relevance (mRMR) to lessen the gene expression of cancer datasets. Next, these subsets of genes are classified using deep learning (DL) to identify different groups or classes related to a particular cancer disease. Eight different benchmark microarray gene expression datasets of cancer have been utilized to analyze the performance of the proposed approach with different evaluation matrix such as recall, precision, F1-score, and confusion matrix. The proposed gene selection method with DL achieves much better classification accuracy than other existing DL and machine learning classification models with all large gene expression dataset of cancer.

## Introduction

**Fig. 1:**
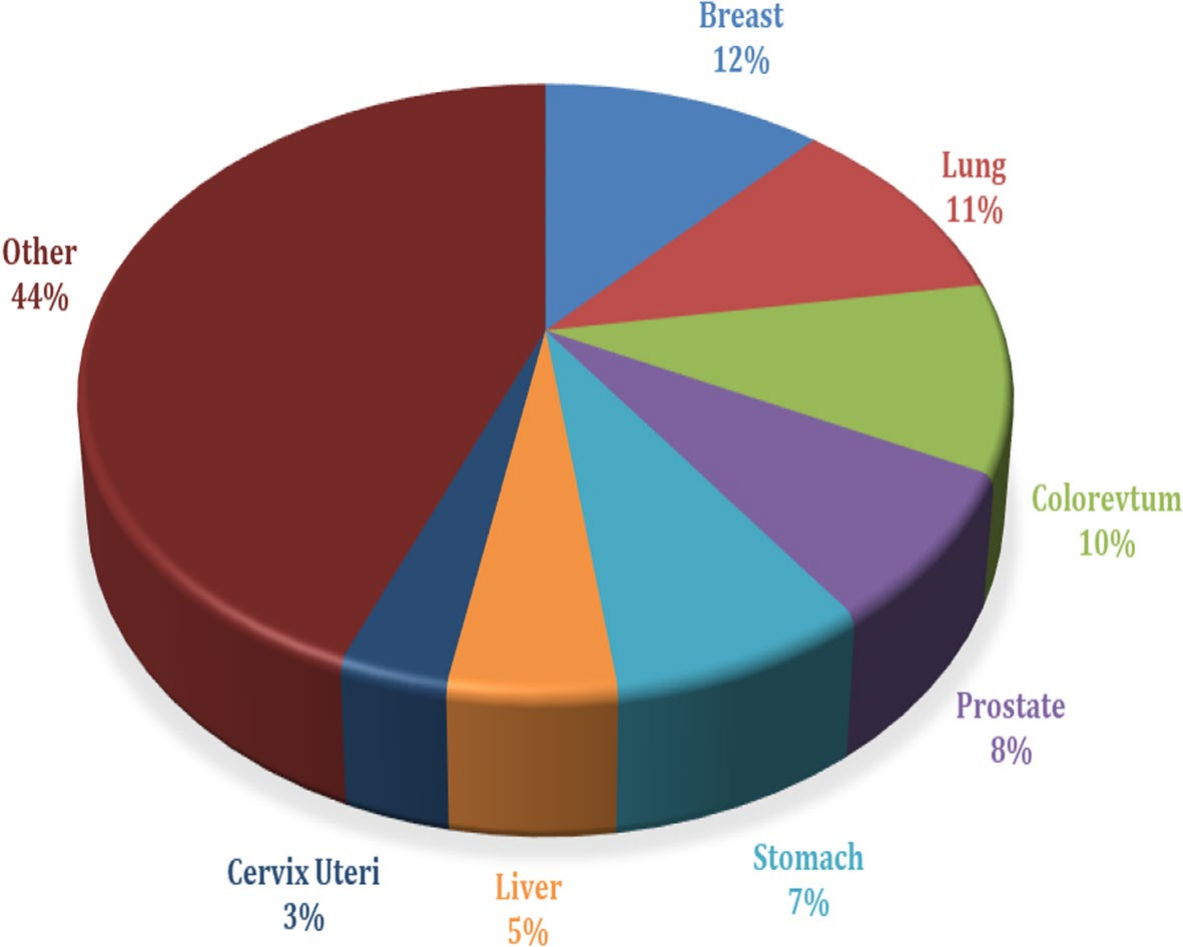
Estimated number of new cases in 2023, worldwide with both sexes and all ages

Successful cancer therapy has remained a significant issue despite enormous improvements in healthcare over the past century, and it is the second leading cause of mortality globally, after cardiovascular disease [1]. According to data from the World Health Organization (WHO), cancer is the leading cause of death worldwide. Of the estimated 18.1 million cancer cases worldwide, 9.3 million cases involved males and 8.8 million involved women. The most common types of cancer are lung, liver, prostate, colon, breast, and rectum [1]. Figure 1 illustrates the projected worldwide count of new cases, categorized by age groups and gender based on 2023 estimates delivered by the American Cancer Society (ACS) [1, 2]. Clinical research and the treatment of many diseases are significantly influenced by the gene expression levels in an organism [3]. Gene expression microarray data is also known as gene-chip is a scientific advanced tool used by many researchers to study the magnitudes of several genes expressed in the abnormal sample [4]. It serves as a tool that reflects the possible spectrum of the genome to analyze and investigate the root cause of the diseases. Problems related to gene expression profile could be solved using DNA microarray and RNA-seq based platform [5]. The use of gene expression profile in genetic research is a potent strategy that presents the data scientist with several analytical difficulties [5]. In order to locate the relevant gene that is conveyed, advanced biomarker machine learning approaches help by using gene expression data [6]. The development of trustworthy cancer biomarkers is crucial for the field of clinical diagnostics [6]. Gene expression profiles like microarray technology and RNA-seq based platforms with machine learning and deep learning are useful in managing and isolating the genes responsible for inherited diseases [7, 8]. It helps to design suitable treatments in suppressing the magnitude of expressed genes linked with inherited diseases during the early development of the organism. The gene expression profiles generate high dimensional data, which is a major issue to deal with before creating the actual classifier. The accuracy and cost of computation affect the performance of the classifier [7]. The specific methods to decrease the dimensionality of the gene expression and to conquer the related problems are the Feature selection technique & method of Feature extraction [8]. The latter provides new fewer size features, condensing the properties of high dimensional features as far as possible and the previous, on the other hand feature selection, filters irrelevant and reductant features and includes critical informative features [9]. The optimization techniques of linear algebra and the core part of statistics are the fundamental tools of most of the machine algorithms developed for gene expression data analysis [10]. Analyzing the expression patterns of genes can be approached through diverse machine learning methodologies [11, 12]. However, the efficacy and precision of deep learning (DL) algorithms in this context have garnered significant attention due to their capacity for capturing complex patterns and dependencies inherent in molecular interactions [13, 14]. This precision makes DL is a valuable tool for advancing our understanding of gene expression in various biological processes. Early screening for cancer is important before they damage vital organs, as it is very difficult to treat once it invades and most cancers have a moderately high chance of being cured if diagnosed and treated at early stages [15]. Hence, early prediction of cancers plays vital role in clinical management of the disease. Researchers leveraging advanced computational models to analyze intricate patterns and subtle indicators within diverse datasets, contributing to more accurate and timely cancer prognosis.

Salem et al. implemented Information Gain & Standard Genetic Algorithm to classify human cancer disease depending on gene expression profiles. The Information Gain algorithm serves the purpose for feature selection followed by feature reduction and cancer type classification is achieved through hybrid Genetic algorithm and Genetic programming algorithm respectively to improved the accuracy of the classifier [15]. Wang et al. classified microarray data of leukaemia and colon cancer, using the hybrid technique with Adaptive Elastic Net with Conditional Mutual Information. The proposed hybrid algorithm dominates traditional methods not only by improving the accuracy but also by using the minimum number of genes [16]. Medjahed et al. developed a unique two steps algorithm. It is based on Support Vector Machine Recursive Feature Elimination to extract the genes and the latest Binary Dragonfly Algorithm to improve performance of the previous. Authors, for the first time, incorporated the application of the metaheuristics algorithm with microarray data analysis that enhanced the accuracy of the classifier with a minimum number of genes [17]. Jansi et al. implemented two-stage algorithms based on Mutual Information Genetic Algorithm. Screening of potential genes with high mutual values is followed by creating an optimal set of genes through Genetic Algorithm and SVM (Support Vector Machine). The proposed method shows improvement in accuracy when applied on datasets of different types of cancers [18]. Rouhi et al. proposed a hybrid approach which initially reduces the dimension of the features followed by implementation of Advanced Binary Ant Colony meta-heuristic algorithm. The constructed hybrid approach enhanced the accuracy of the classifier when compared with available methods [19]. Venkataramana et al. implemented Parallelized hybrid feature selection (HFS) method. It not only incorporates the statistics related to subsets of features but also ranks them to set the selection of most effective, informative genes. The proposed method established the accuracy of 97% on the data sets related to gastric cancer and improved the accuracy to some extent when compared with available methods [20].

In recent times, Various researchers have employed deep learning classifiers for the classification of microarray data, especially in the context of cancer prediction [21]. Tabares et al. have shown comparative studies on the 11-tumor database and recorded accuracies of 90.6% & 94.43% respectively on logistic regression and convolutional neural networks. The proposed algorithm based on deep learning methods shows more promising results on microarray data analysis [22]. Liu et al. proposed Sample Expansion Based technique with deep learning approaches used for categorization of microarray data. The authors claimed improvement in the accuracy of the classifier after testing the data with proposed algorithms [23]. Zeebaree et al. tackled the main challenges of the classification of cancer microarray data with the help of deep learning algorithms based on Convolutional Neural Network (CNN), which show improvement in accuracy and extraction of informative genes as compared to machine learning model [24]. Aziz et al. evaluates the effectiveness of an Artificial Neural Network (ANN) classifier with six hybrid feature selection techniques, incorporating Independent Component Analysis (ICA) and bio-inspired algorithms for optimization. The study, achieved high classification accuracy with a minimized number of selected genes. Statistical hypothesis testing confirms the significant differences between the algorithms, emphasizing the effectiveness of the proposed approach [21].

Metaheuristic algorithms have emerged as effective solutions for feature selection problems, providing more accurate results [9, 10]. Currently, the Cuckoo Search Algorithm has shown particular promise across various domains, demonstrating its efficacy in addressing feature selection challenges. Alzaqebah et al. presented a study demonstrating use of cuckoo search methods for feature selection. This study involved use of cuckoo search alongside a memory-based mechanism to save optimal solutions (feature vectors) to find features that enhanced the classification accuracy [25]. Swathypriyadharsini et al. have put out a methodology for identifying co-expressed genes that combines tri-clustering methods with a hybridized CSA algorithm and clonal selection. After that, to ascertain the biological importance of the genes in the generated clusters, this technique makes use of gene ontology, functional annotation, and transcription factor binding site analysis. In comparison to both conventional cuckoo search techniques and other current tri-clustering algorithms, the experimental results of this approach were shown to be superior [26]. Zhao et al. proposed a new search algorithm namely, the Elite Hybrid Binary Cuckoo Search algorithm which employed feature weighting and elite strategy to improve over Cuckoo Search. The proposed algorithm showed results outperforming binary genetic algorithm and binary particle swarm optimization algorithm in terms of standard deviation, sensitivity, specificity, precision, and F-measure [27]. Othman et al. use of innovative operators for genomic selection is included in a hybrid multi-objective CSA that has been developed. To do this, this study employed single crossover and double mutation operators. Using seven high dimensional cancer microarray datasets that are freely available, the suggested method was assessed. According to the experimental findings, the suggested technique selected fewer relevant genes while outperforming multi-objective cuckoo search and classic cuckoo search algorithms in terms of performance [28]. Scaria et al. proposed a user-friendly rule-based classification model for processing microarray gene data. Here, cuckoo search optimization algorithm was used to form classification rules and pruned by associative rule mining. This study concluded that the performance of the proposed approach was adequate enough in terms of accuracy, sensitivity, specificity and time consumption [29]. Aziz et al. explored a novel metaheuristic CO-WOA for accurate species identification due to diverse seafood diseases. Performance comparisons with Convolutional Neural Networks (CNN) and VGG-19 validate the proposed method's applicability, showcasing 100% accuracy in the suggested deep learning model. The study outperforms other models like ResNet150V2, DenseNet, Visual Geometry Group-19, Inception V3, and Xception, establishing the Proposed Deep Learning model as the most effective through empirical analysis leveraging artificial neural networks [30].

**Fig. 2:**
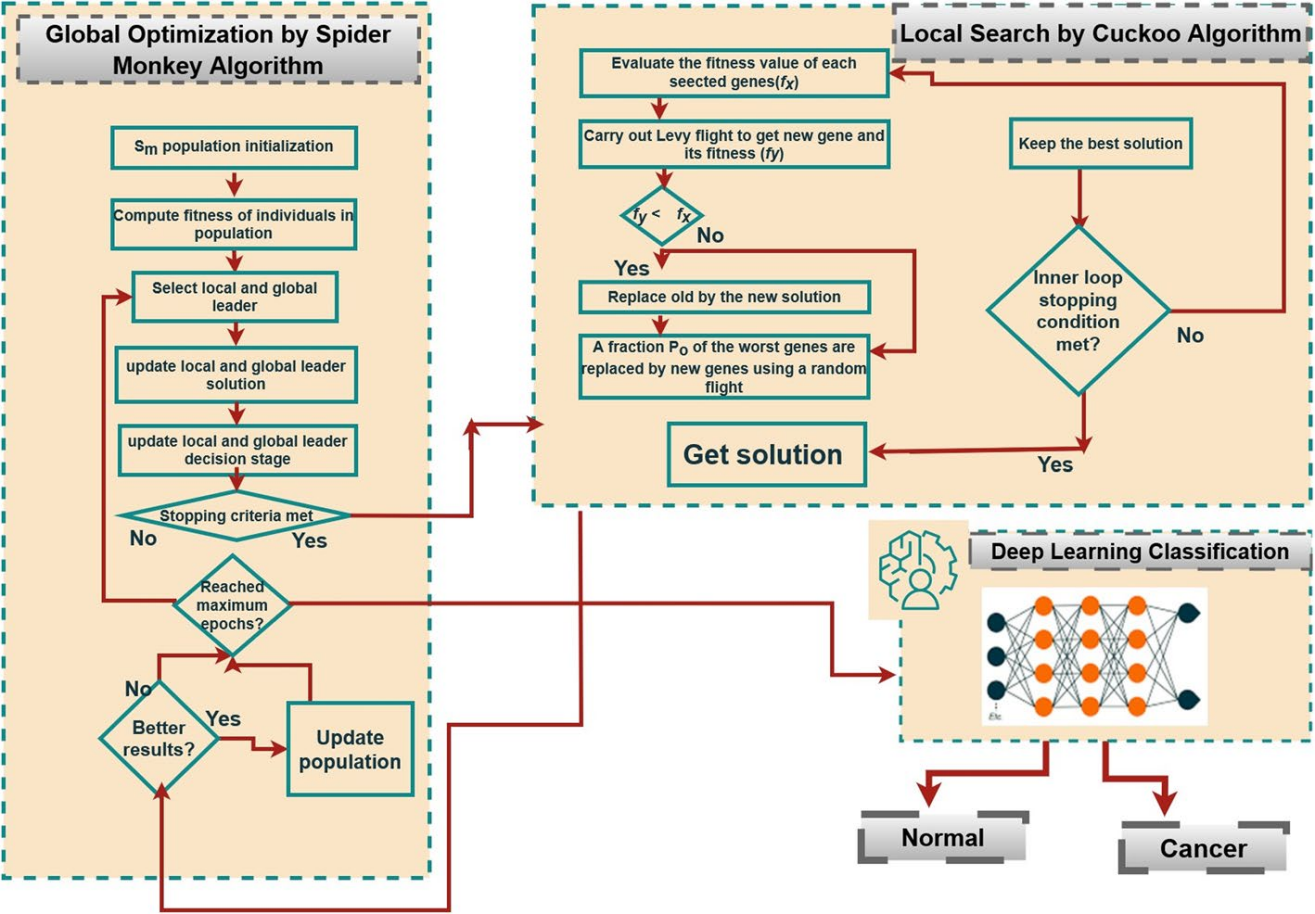
The framework of the proposed model

The important findings of this work defined as:

- Hybrid metaheuristic learning-based approach has been designed with DL classifier for gene selection that classify cancer accurately using SMO and CSA as CSSMO to optimize the selected genes even if the patients are in an early stage.
- Enhance the CSSMO results by adopting filtering method mRMR, to reduce the dimensionality of gene expression data.
- The result of deep learning model with proposed hybrid approach achieves much better accuracy than other existing DL models. Figure 2 shows the complete framework of the proposed model.

This paper focuses on identifying compact gene groups using CSSMO for efficient deep-learning classification of cancer classes. The remainder sections of the research document are arranged as follows: "Materials and methods" section presents initial learning terminology related to CSA, SM algorithms and DL and the proposed CSA and SM based important feature identification algorithm. In "Experimental setup" section explained complete experimental setup and parameter setting of proposed algorithm. "Experimental results and discussion" section outlines the empirical evaluation and gives outcome. Finally, “Output the final optimized solution with the below three steps” section summarizes our paper.

## Materials and methods

### Deep learning

Deep learning, a specialized domain within the broader landscape of machine learning [13, 14]. DNNs have algorithm in the field of become the gold standard computer vision, achieved this by bestowing computers with the remarkable capacity to autonomously acquire and discern intricate patterns present within expansive and complex datasets, thereby emulating the intricate neural networks observed within the human cerebral architecture [31]. Optimization of such DNNs helps to improvise the classification results, backpropagation is one such approach [32]. The backpropagation method, an optimization technique integral to the field of deep learning, operates as a vital component within each localized segment of a CNN [33]. This algorithm assumes a pivotal role by meticulously fine-tuning the network's parameters through iterative computations of gradients associated with an objective function, consequently facilitating the localized optimization process [23, 33]. This technique has become indispensable in the optimization of deep neural networks, allowing them to attain exceptional levels of predictive accuracy across diverse and high-dimensional datasets [14, 30].

### Cuckoo search algorithm (CSA)

CSA, pioneered by Yang and Deb in 2009, stands as a population-based metaheuristic optimization paradigm. Its genesis finds inspiration in the intriguing reproductive behaviour of cuckoo birds, characterized by their clandestine practice of laying eggs in the nests of unsuspecting host bird species, entrusting them with the incubation and care of their progeny [34]. In the field of optimization, CSA embarks on a meticulous traversal of the solution space, with the overarching goal of unearthing the most optimal solution to a given problem [35, 36]. This optimization unfolds through a sequence of intricately choreographed phases encompassing reproduction, selection, and replacement mechanisms [37]. Within the computational framework, each solution assumes the guise of a symbolic cuckoo egg, signifying a potential resolution to the optimization conundrum at hand [38]. The odyssey commences with the stochastic creation of a population of these virtual cuckoo eggs [39]. As the quest progresses, select cuckoo eggs undergo replacement, being supplanted by novel solutions engendered through a stochastic random walk process, an analogue to the reproductive strategies of the avian inspiration [27, 28, 38]. To further augment its exploratory prowess, CSA integrates a Levy flight strategy, orchestrating the construction of fresh solutions designed to liberate the algorithm from local optima entrapment, thus facilitating a more comprehensive traversal of the solution [25, 26, 39].

### Spider monkey optimization (SMO) algorithm

The SMO (Spider Monkey Optimization) algorithm stands as a member of the swarm intelligence domain within metaheuristic optimization techniques [40]. Drawing inspiration from the foraging behaviour of spider monkeys, it orchestrates a collective effort among a population of solutions, akin to a group of spider monkeys, in the pursuit of an optimal solution [40, 41]. This pursuit involves the dynamic exchange of information among the individuals as they continuously refine their positions during the iterative optimization process [41]. This algorithm operates through a structured sequence of six distinct phases meticulously designed to enhance the solution positions while mitigating the risks of stagnation or premature convergence [41, 42]. Commencing with the assignment of initial positions, randomly generated for each solution, it proceeds to refine these positions iteratively [42]. Within the population, the most exemplary solution is accorded the title of the global leader, while the algorithm also has mechanisms for grouping individuals if the global leader's performance plateaus over a certain number of iterations [43]. Each group then features its local leader, representing the best solution within that specific subgroup [41, 43]. Moreover, the algorithm integrates phases for generating trial solution positions, the selection of both global and local leaders, and strategies for addressing stagnation and premature convergence issues at both the population and group levels [41, 43]. Through this intricate dance of information sharing and position refinement, the SMO algorithm orchestrates a collective intelligence strategy inspired by the food-finding prowess of spider monkeys to navigate complex optimization landscapes effectively [41, 42]. The algorithm might exhibit weaknesses in striking the right balance between exploration and exploitation [40]. Specifically, it might struggle with local optima traps, where it becomes entrenched in suboptimal solutions due to its exploration-centric nature [43]. This limitation can hinder its ability to efficiently exploit promising areas of the search space [44].

### Proposed methodology CSSMO

CSSMO (Cuckoo Search and Spider Monkey Optimization) has been proposed, this algorithm seamlessly integrating the strengths of two prominent metaheuristic algorithms: CSA and SMO, to enhance solution discovery [36, 45]. This method comprises three distinctive phases: an initial preprocessing phase, followed by the application of Cuckoo Search, and a Spider Monkey-based feature selection strategy. Finally, it culminates in the classification of cancer utilizing a selection of genes optimized through CSSMO, employing Deep Learning classifiers for precise diagnostic outcomes. "This hybrid approach is rooted in a referenced framework that adeptly manages the intricate balance between exploration and exploitation, thereby enhancing optimization efficacy, particularly in complex problem spaces [36, 44–46]. The cited reference provides foundational insights into the integration of these two strategies, ensuring a nuanced and effective approach to addressing complex optimization challenges."

#### Preprocessing phase

Gene expression datasets pose a significant challenge because they contain a lot of genetic information from many genes. If we use these datasets without any preparation, it can slow down our algorithm and make it less accurate. It also complicates the process of classifying the data. To tackle these issues, we've added the mRMR method as a crucial step before we start working with the data. The main goal of using mRMR is two-fold: first, it helps us get rid of unnecessary information and reduces the number of repetitive genes [47]. This makes our cancer classification model work better and give more accurate results. It does this by looking at two important things: first, it checks how related genes are to different types of cancer, and second, it figures out if some genes are very similar to each other [47]. Using mRMR before we start our work helps us choose the most important genes for predicting cancer and removes any unimportant data. This makes our CSSMO algorithm work better and gives us more reliable result and compute redundancy respectively.

#### Feature selection phase (CSSMO algorithm)

The domain of nature-inspired metaheuristic optimization techniques in scholarly literature underscores their accomplished history in addressing a wide spectrum of challenges [9, 30]. However, it is crucial to recognize that each algorithm possesses distinctive attributes and limitations, rendering them suitable for particular optimization scenarios [10]. In the domain of microarray data feature selection, replete with numerous variables and combinatorial complexities, an array of soft computing approaches has been explored [8]. The essence of the matter lies in methodically evaluating the performance of these algorithms and identifying the one that aligns most favourably with the unique requisites of a given problem [12]. In this vein, our study introduces a hybrid metaheuristic methodology that capitalizes on the complementary characteristics of CSA and SMO algorithms to pinpoint optimal solutions for intricate optimization tasks. CSA excels in localized search capabilities, characterized by a reduced number of control parameters and a compact population size [36]. Conversely, the SMO algorithm specializes in global search and demonstrates resilience, although it can be susceptible to early convergence and slower convergence rates relative to alternative methodologies [44, 45]. Our innovative hybrid approach strategically harnesses the strengths of both algorithms by replacing the local fitness phase of the SMO algorithm with the local fitness mechanism derived from the CSA algorithm. This integration, denoted as the CSSMO algorithm, is designed to heighten the efficiency and efficacy of the optimization endeavour, streamlining the pursuit of optimal solutions.

**Pseudo Code:** Hybrid (CSSMO) Algorithm:

1. Initialize the algorithm population, control parameters ($LocalLeaderLimit$ & $GlobalLeaderLimit$) and Perturbation rate ($pr$).
2. Calculate fitness metrics (i.e., distance of population individuals from the food source).
3. Select global leader via greedy selection based on fitness metrics and use cuckoo search optimization for local leader selection.
4. Repeat the following steps until the termination criteria is not met:
  - Position update for all individuals in the population based on Local Leader Phase (LLP) by using self-experience, local leader experience, and group member experience.
  - Greedily select newly generated positions based on fitness metrics.
  - Calculate probability (${prob}_{i}$) using equation.
  - Position update for all group members selected by ${prob}_{i}$, based on Global Leader Phase (GLP) by using self-experience, global leader experience, and group member experience.
  - Update the position of the local and global leaders by applying greedy selection.
  - If control parameters bind a Local Group Leader, redirect all members in that group for foraging using Local Leader Phase Optimized with Cuckoo Search.
  - If control parameters bind a Global Leader, divide the group into smaller groups using Global Leader Phase (GLDP).
5. Output the final optimized solution with the below three steps:

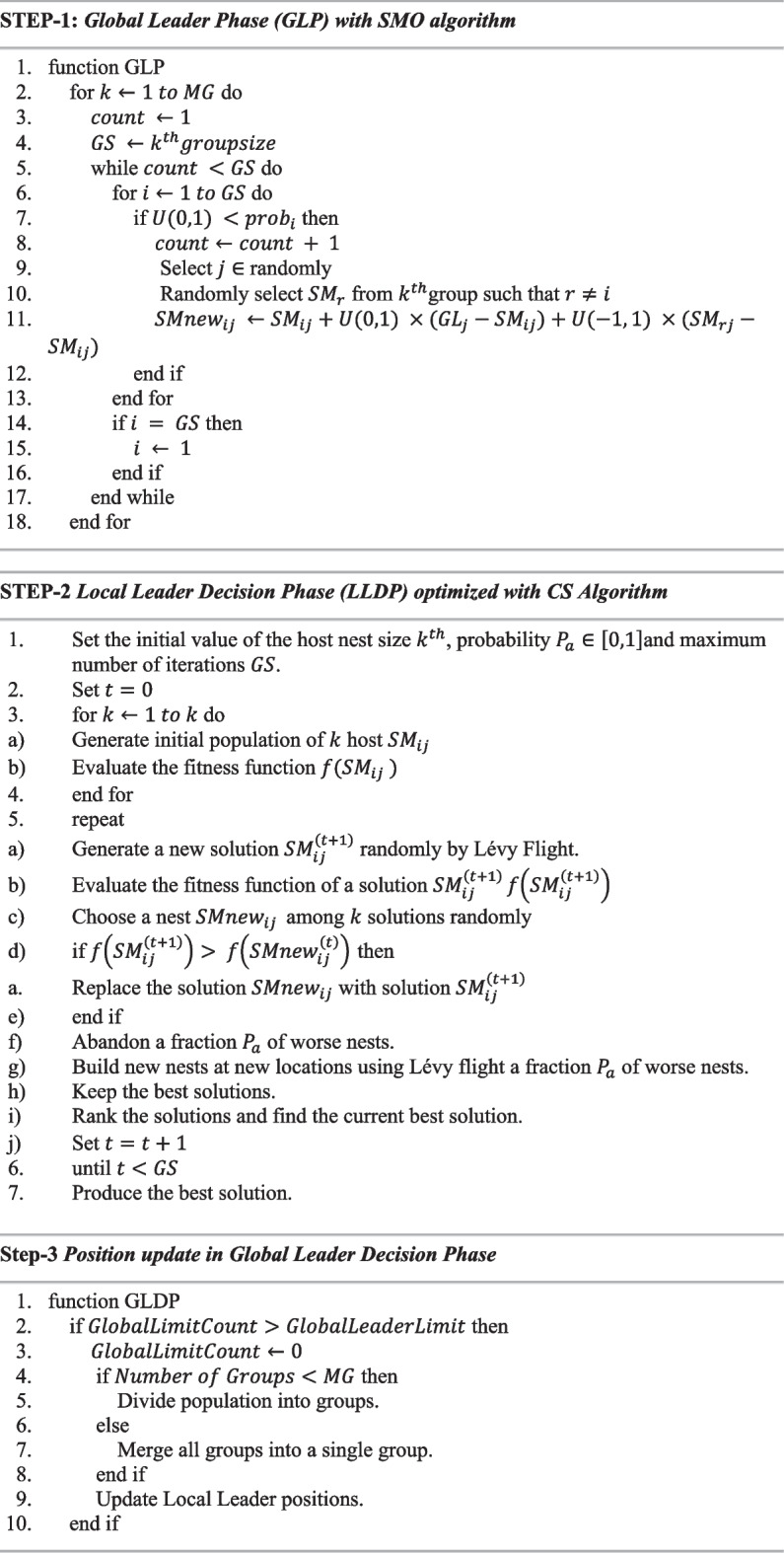


## Experimental setup

In this research, we employed CSSMO as a optimization technique to optimize the best subset of gene that selected by mRMR method, which served as inputs for the DL classification. The experimentation was performed using the Operating System Ubuntu 20.04.5 LTS (Windows WSL) with IDE VS Code (Python) platform on a computer system that featured an Processor Intel(R) Core ™ i9-12900 k (5.20 GHz) and 64 GB of RAM with Nvidia RTX Quadro A5000 Graphics Processing Unit (GPU).

### Dataset used

Experiments were carried out to determine the efficiency of our technique. To assess the proposed algorithm's accuracy, we used eight benchmark data sets: Leukemia, Colon, Prostate, Lung Cancer 2, Leukemia 2, and High-Grade Glioma. The characteristics of these datasets are described in Table 1. In the course of this research, we employed multiple datasets to substantiate our research objectives. All the utilized datasets are accessible through the following link: https://csse.szu.edu.cn/staff/zhuzx/Datasets.html.

**Table 1:** Detail of eight cancer microarray data

Data set	Number of classes	Number of genes	Class balance ±	Number of samples	Brief description
Colon cancer [48]	2	2000	(22\40)	62	Colon cancer data gathered from patients who had tumor biopsies reveal that both routine positive biopsies and negative tumors come from healthy portions of the same patients' colons
Acute leukemia [49]	2	7129	(47\25)	72	Acute Leukemia consists of two categories: category 1 is the Acute Myeloid Leukemia (AML) with 47 samples and category 2 is Lymphoblastic Leukemia (ALL) with 25
Prostate tumor [50]	2	12,600	(50\52)	102	Prostate tumor data was acquired from two types of samples: non-tumor (normal) and tumor samples (cancer)
High-grade Glioma [51]	2	12,625	(28\22)	50	High-grade Glioma contains glioblastomas and anaplastic oligodendrogliomas from brain tumor tissues
Lung cancer II [52]	2	12,533	(31\150)	181	Lung cancer II comprises of Malignant Pleural Mesothelioma (MPM) and Adenocarcinoma (ADCA) tissue samples of the lung
Leukemia 2 [53]	3	7129	(28\24\20)	72	The Leukemia 2 data set includes three types of samples: 28 AML samples, 24 ALL samples, and 20 MLL samples
Breast [54]	2	24,481	(51\46)	97	Breast cancer data include two type of samples: non-relapse 51 samples and relapse 46 samples
Ovarian [55]	2	15,154	(162\91)	253	Ovarian cancer data include 162 cancer samples and normal 91 samples

### Deep learning model configuration

**Fig. 3:**
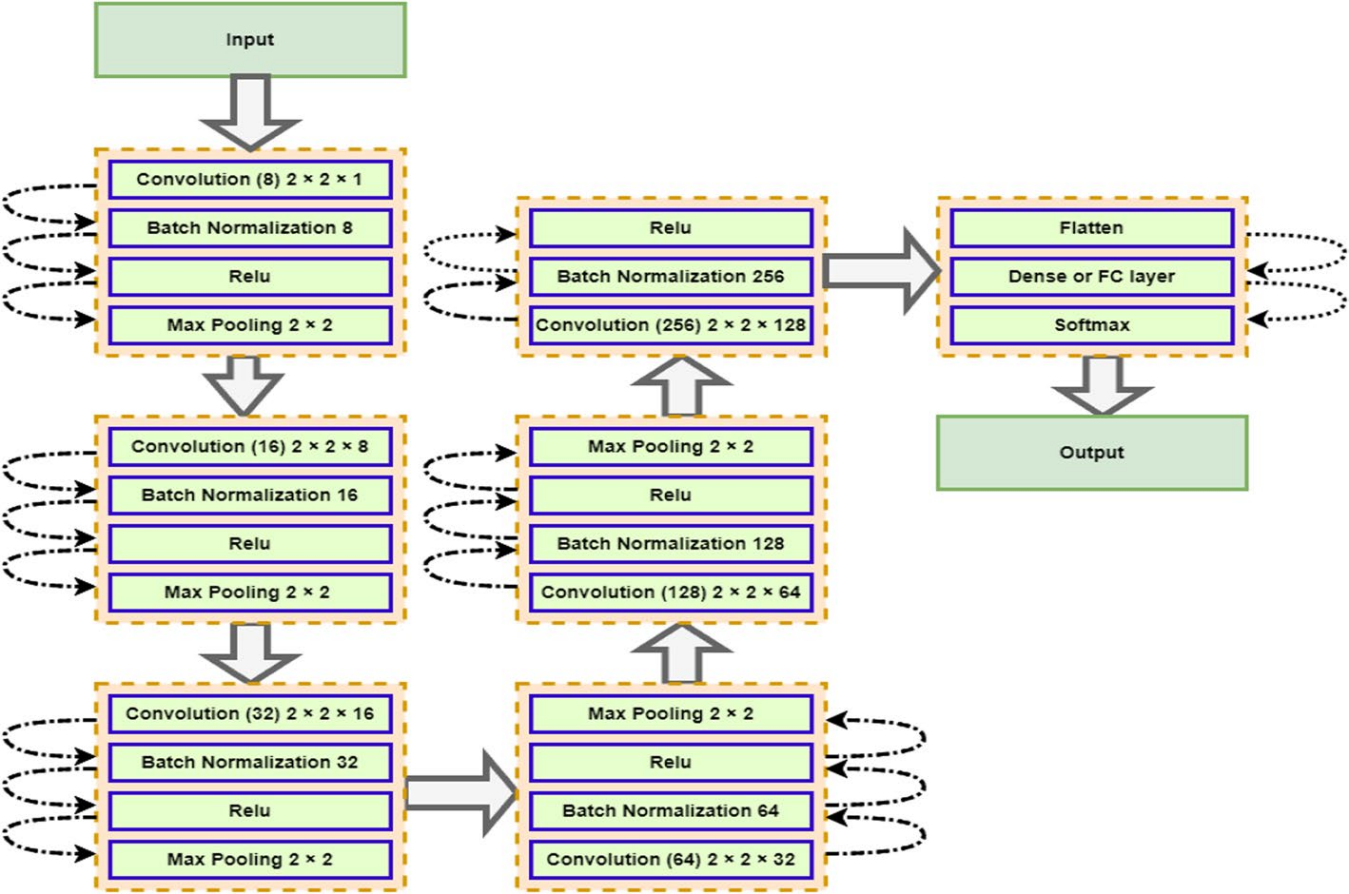
Visualization of used deep learning model configuration

Figure 3 depicts a deep learning model configuration that consists of eight convolutional layers. The first layer, "Convolution 8 2 × 2 × 1", applies 8 filters of size 2 × 2 to the input data, with a stride of 1. The second layer, "Convolution 16 2 × 2 × 8", applies 16 filters of size 2 × 2 to the output of the first layer, with a stride of 1, and uses 8 as the number of input channels. Similarly, the third layer, "Convolution 32 2 × 2 × 16", applies 32 filters of size 2 × 2 to the output of the second layer, with a stride of 1, and uses 16 as the number of input channels. The fourth layer, "Convolution 64 2 × 2 × 32", applies 64 filters of size 2 × 2 to the output of the third layer, with a stride of 1, and uses 32 as the number of input channels. The fifth layer, "Convolution 128 2 × 2 × 64", applies 128 filters of size 2 × 2 to the output of the fourth layer, with a stride of 1, and uses 64 as the number of input channels. The last layer, "Convolution 256 2 × 2 × 128", applies 256 filters of size 2 × 2 to the output of the fifth layer, with a stride of 1, and uses 128 as the number of input channels. ReLU (Rectified Linear Unit) is a commonly used activation function in neural networks. It applies an operation on each element of the input, where any element less than zero is set to zero and any element greater than zero is passed through unchanged. This operation is defined mathematically as $y=(0, x)$, where $x$ is the input and $y$ is the output. This function allows the network to converge faster and reduces the chances of encountering the vanishing gradient problem, as it increases the network's non-linearity. Max pooling is a technique used to down-sample the spatial dimensions of the input data, typically used after the convolutional layer in CNN. The max pooling operation is applied to small rectangular regions of the input data, called pooling windows, and for each window the maximum value within that window is selected and propagated to the next layer.

This operation helps to reduce the number of parameters in the network, reduce overfitting and preserves the dominant features in the images. Batch normalization is a technique used to normalize the input layer by adjusting and scaling the activations. The idea behind this technique is to ensure that the inputs of each layer are in the same distribution and thus accelerate the convergence of the network. It normalizes the input data by re-centering and re-scaling them so that the mean of the data is zero and the standard deviation is one. During training, it maintains a moving average of the mean and variance of the data and during testing it uses these values to normalize the test data. This allows the network to be less sensitive to the initial conditions of the parameters, reducing the need for careful parameter initialization and makes it possible to use much larger learning rates, which speeds up the training process. Based on the above discussion, in our model between each of the convolutional layers, there is a batch normalization operation and a ReLU activation function which serves as a non-linearity to the output of the convolution operation. The output of each batch normalization and ReLU operation is then passed through a max pooling operation, except for the last layer, which does not have max pooling applied.

### Parameter setting of proposed method

The fitness function given here is used to assess the accuracy of the proposed model. It is used to assess how well the model's output matches the predicted or actual outcomes.

1 $$Accuracy = \frac{CC}{N} \times 100$$

Equation 1 refers to the fitness function of the proposed approach, which is used to evaluate the classifier's performance. The fitness function is dependent on the classifier's prediction accuracy, which is a measure of how successfully the classifier categorizes data. In the equation, N is the total number of samples in the relevant class, and CC is the number of properly classified observations. The number of correctly classified observations is the numerator of the equation, while the total number of samples in the class is the denominator. The accuracy is the resultant number, which ranges from 0 to 1, with 1 indicating perfect accuracy and 0 indicating no accuracy. Finally,

2 $$Fitness\left( f \right) = Accuracy \left( {f_{a} } \right)$$

The LOOCV accuracy has been utilized as a fitness function to evaluate the classifier's performance. It is critical to grasp the parameters and their values in order to properly comprehend the performance of the suggested approach. It’s also worth mentioning that alternative parameter setups may be required for different issue domains. Algorithm 1 illustrated the all-sequential steps of modified proposed algorithm and Table 2 shows the parameters used for the proposed algorithm.

**Table 2:** Parameter setting of the proposed CSSMO algorithm

Parameter	Value
Number of nests (population)	50
Total No. of eggs	10
Total No. of generations	200
Minimum probability ${(P}_{\alpha })$ of discovering an egg ${P}_{{a}_{min}}$	0.3
Maximum probability ${(P}_{\alpha })$ of discovering an egg ${P}_{{a}_{max}}$	0.5
$\alpha$ Step size	1
The swarm size N	50
MG	5
Global leader limit	50
Local leader limit	1500
The number of simulations/runs	100

**Figure:**
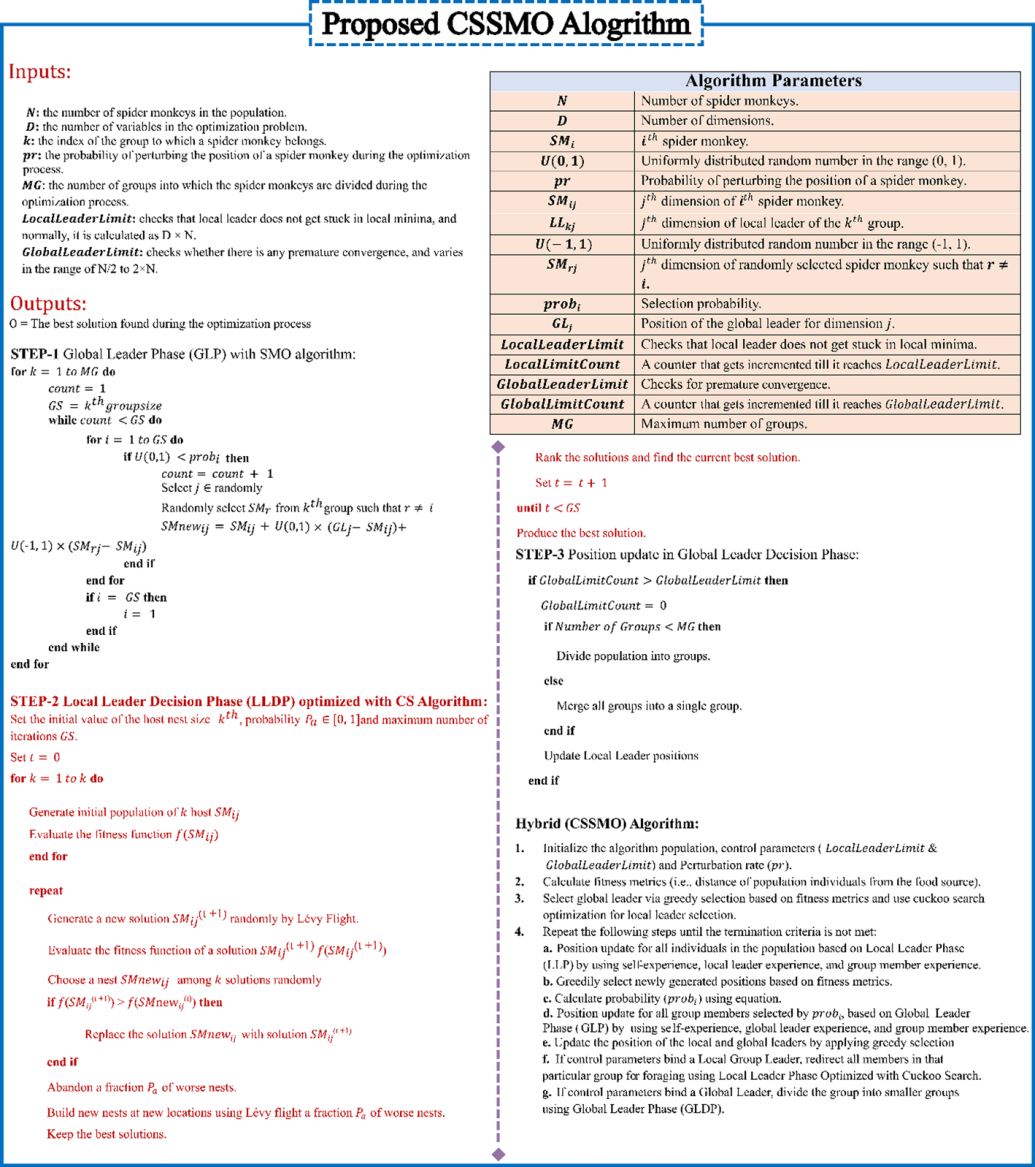
**Algorithm 1**

## Experimental results and discussion

### Deep learning classification accuracy

**Fig. 4:**
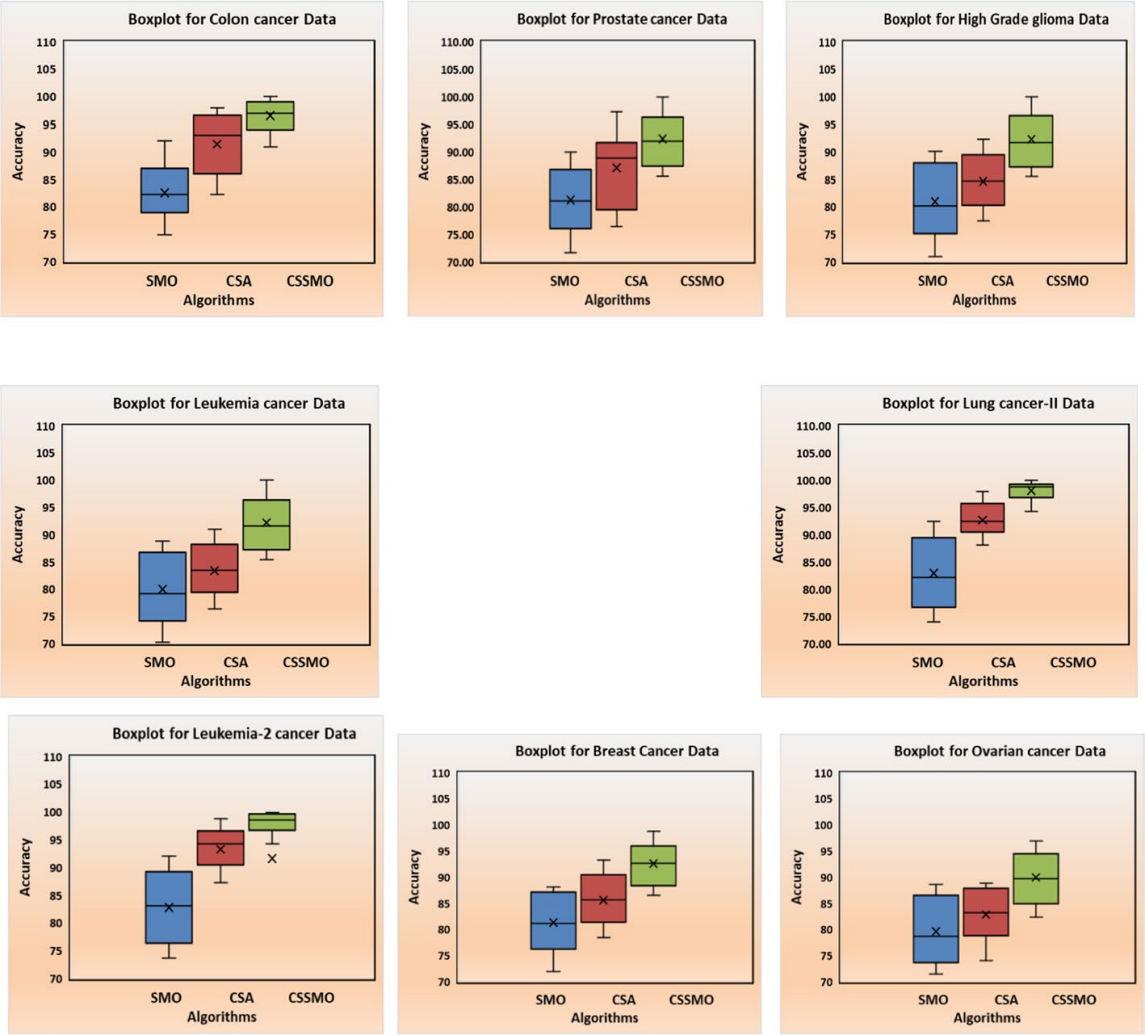
Classification accuracy results with boxplot analysis for the SMO, CSA, and the proposed CSSMO algorithms

Table 3 presents a comparative analysis of classification outcomes with Standard Deviation (SD), revealing that the CSSMO algorithm consistently achieves higher accuracy than the CSA and SMO algorithms across various datasets. Moreover, the comparison is visually depicted through boxplots in Fig. 4. Both the tabulated results and graphical representation affirm that while CSA and SMO algorithms exhibit commendable cancer classification accuracy, the CSSMO algorithm consistently outperforms them, reaching a maximum accuracy of 100% across all eight datasets employed. The box plot in Fig. 4 provides a comprehensive representation of the statistical measures, including mean, maximum, and minimum accuracy, across all eight cancer datasets. It also indicate the convergence exhibited by the CSA, SMO, and CSSMO algorithms.

**Table 3:** Classification accuracy of SMO, CSA, and CSSMO algorithms for all eight data sets

S. No	Mean classification accuracy (CA) and standard deviation (SD)					
	SMO algorithm		CSA algorithm		CSSMO algorithm	
	Mean CA	SD	Mean CA	SD	Mean CA	SD
Colon cancer	88.7	6.02	93.7	5.18	98.9	2.02
Acute leukemia	88.92	8.66	88.67	7.41	98.23	2.12
Prostate tumor	86.99	8.12	89.12	4.37	99.02	1.23
High-grade Glioma	89.21	5.23	87.99	6.21	99.11	1.42
Lung cancer II	90.33	4.22	94.21	2.71	99.51	0.9
Leukemia 2	89.67	3.44	95.44	1.76	100	0.5
Breast data	90.23	4.12	92.44	2.34	98.32	1.56
Ovarian cancer data	91.45	5.06	93.44	3.06	96.98	2.01

### Error estimation

**Fig. 5:**
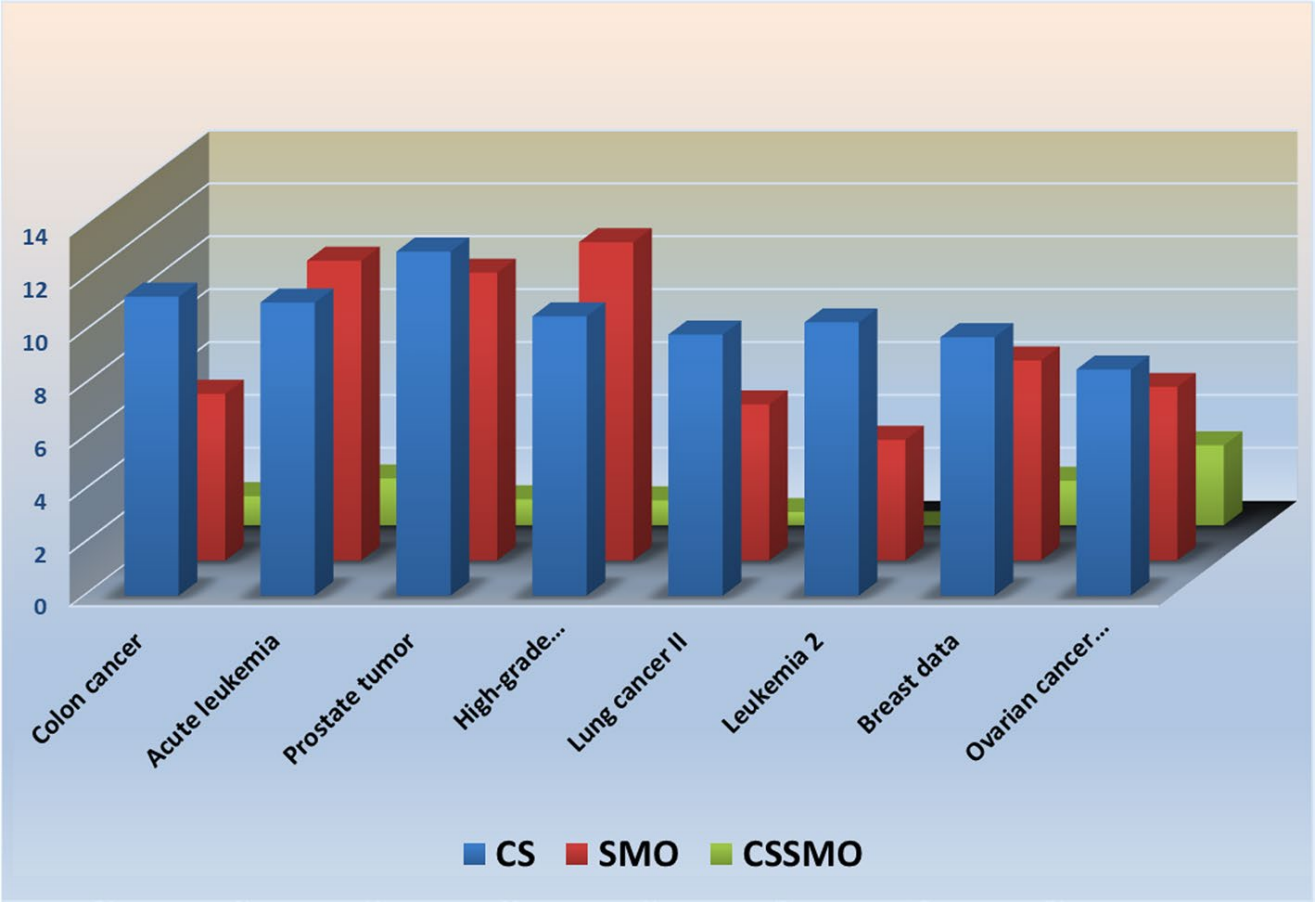
Comparison of average error rate for all 8 cancer datasets with CSA, SMO, and CSSMO algorithms

Figure 5 provides insight into the classification errors of a deep learning model utilizing three different algorithms for all eight datasets. Notably, the CSSMO algorithm consistently outperforms the CS and SMO algorithms across eight cancer datasets. The CSSMO algorithm generally exhibits the lowest prediction errors, showcasing its superior performance compared to the other two algorithms for each dataset.

### Model performance

**Fig. 6:**
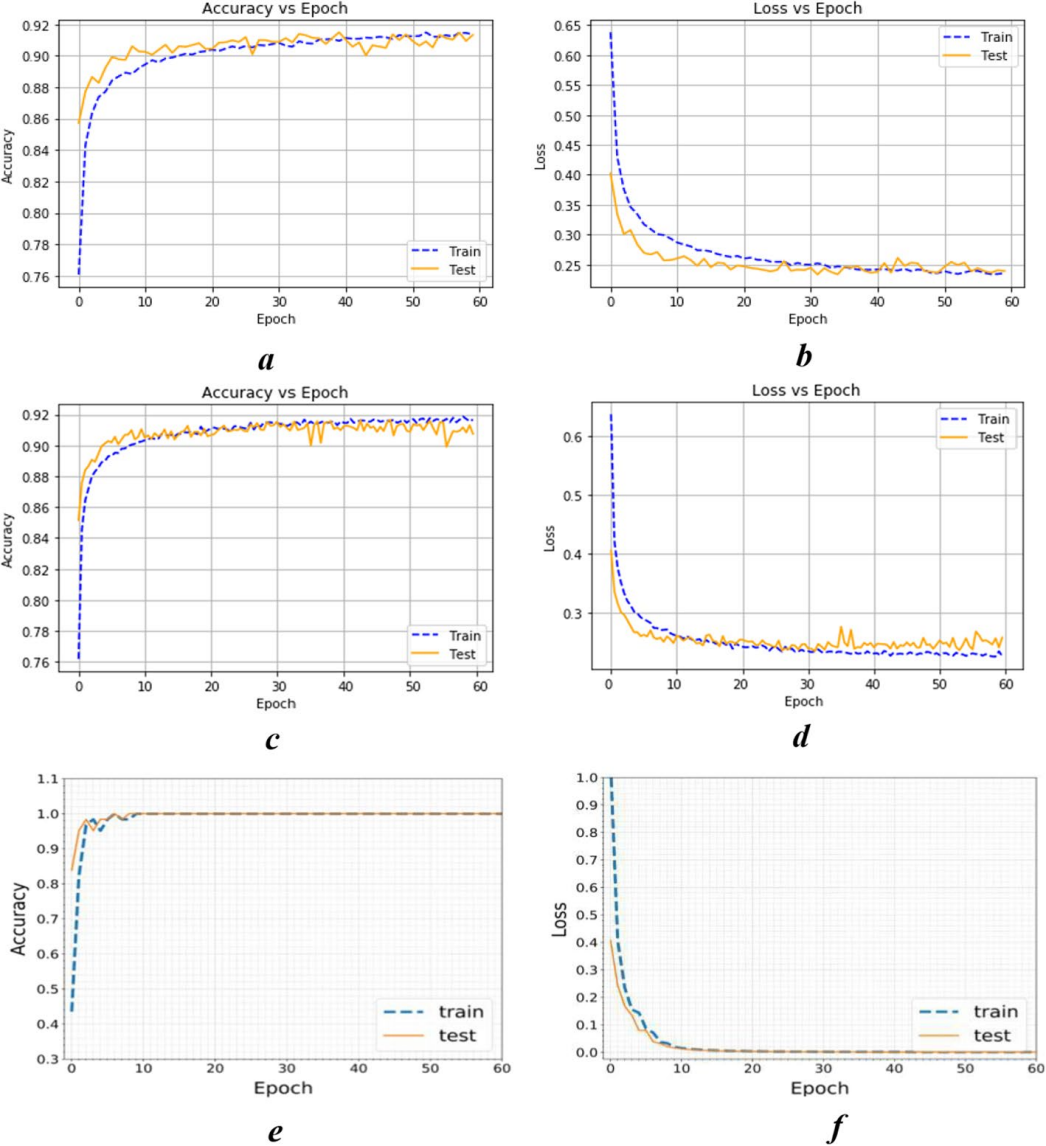
Accuracy versus epochs and loss versus epochs of deep learning for CSA, SMO and CSSMO algorithms

In Fig. 6a–f, the training accuracy and loss scores are used to assess the model's performance on training data. Training accuracy is the proportion of properly categorized instances in the training set, whereas the training loss is the mistake of the model in predicting the right output for a given case. The testing accuracy and loss scores assess the model's ability to generalize to new, previously unknown data. The testing accuracy is the proportion of properly categorized instances in the test set, whereas the testing loss is the model's inaccuracy in predicting the right output for a particular example in the test set.

Figure 6a, b plots accuracy and loss vs epochs for CSA algorithm, it has a relatively large gap between training and testing accuracy and loss. On the other hand, in Fig. 6c, d plots accuracy & loss vs epochs for SMO algorithm which shows a narrower gap between training and testing accuracy and loss. Figure 6e, f plots accuracy & loss vs epochs for CSSMO algorithm, it clearly shows that the CSSMO algorithm has the least difference in accuracy and loss between training and testing compared to CSA and SMO, indicating that hybrid algorithm CSSMO can learn from training data and generalize effectively to new, unknown data. Based on the facts supplied, the CSSMO is the most effective of the three algorithms for reducing gene dimensionality.

### Confusion matrix

**Fig. 7:**
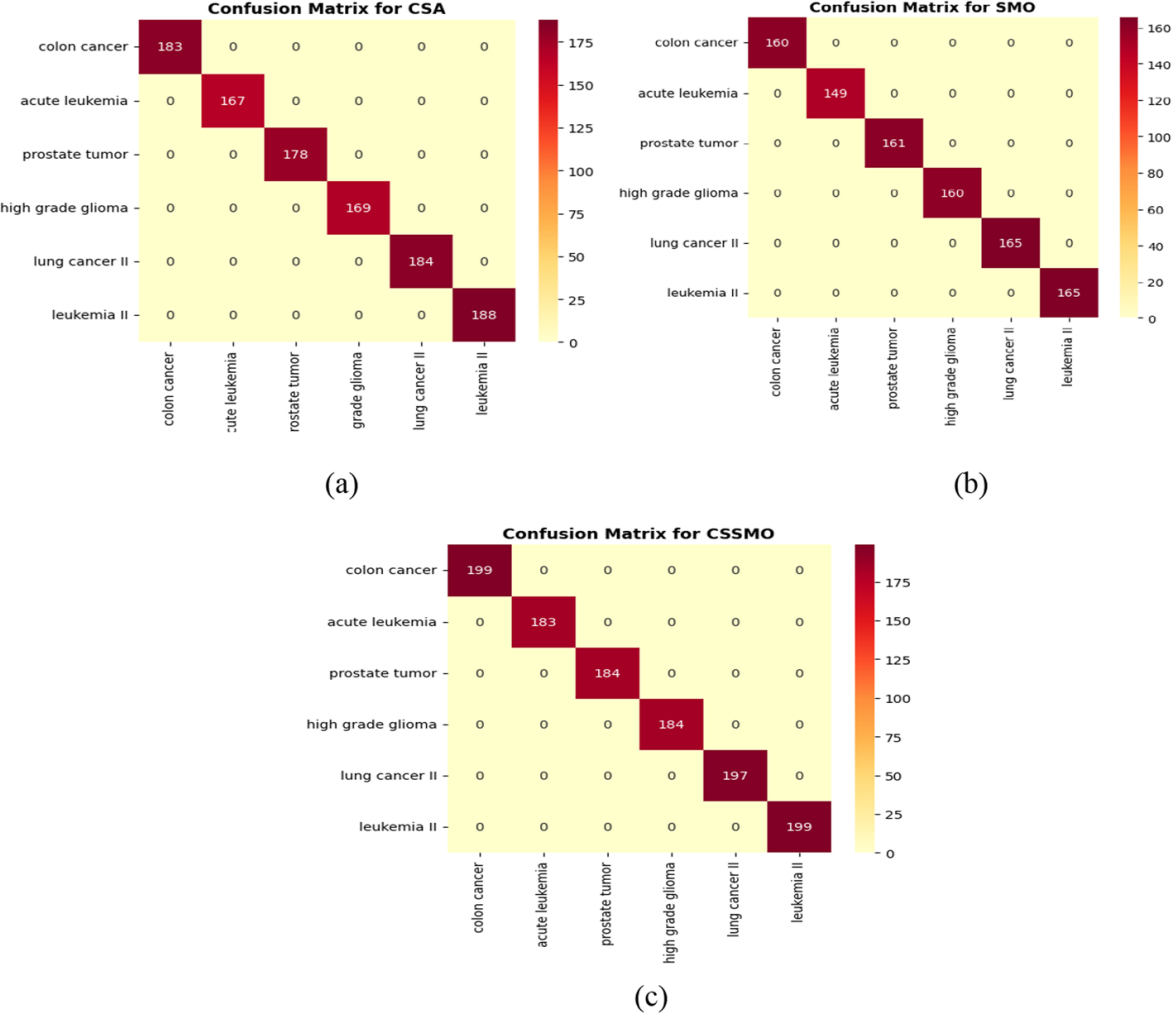
Confusion matrix for **a** CSA, **b** SMO and **c** CSSMO algorithms

In Fig. 7a–c, we have used confusion matrix to evaluate the performance of a classification made by CSA, SMO and the proposed CSSMO algorithm. It is a summary of the actual and predicted class labels for a given set of test data. The rows of the matrix represent the actual class labels, while the columns represent the predicted class labels. In the case of the three algorithms CSA, SMO, and CSSMO, the confusion matrices show the number of correct and incorrect classifications made by each algorithm on a set of test data. The diagonal values of the confusion matrix represent the number of correct classifications made by the algorithm. Figure 7a shows confusion matrix for CSA algorithm, Fig. 7b shows confusion matrix for SMO and lastly Fig. 7c shows confusion matrix for CSSMO algorithm. In Fig. 7c CSSMO algorithm had the highest number of correct classifications on the test data, as indicated by the highest diagonal values in the confusion matrix. This indicates that CSSMO algorithm is the best for classifying the eight different types of cancer and hence the most effective one.

### Comparison with others machine learning and deep learning model

For further comparisons, the proposed algorithm employed with most popular machine learning (SVM and NB classifiers) and deep learning (VGG and LeNet classifiers), being a widely used classifier for medical data classification and cancer classification from gene expression profiles.

**Fig. 8:**
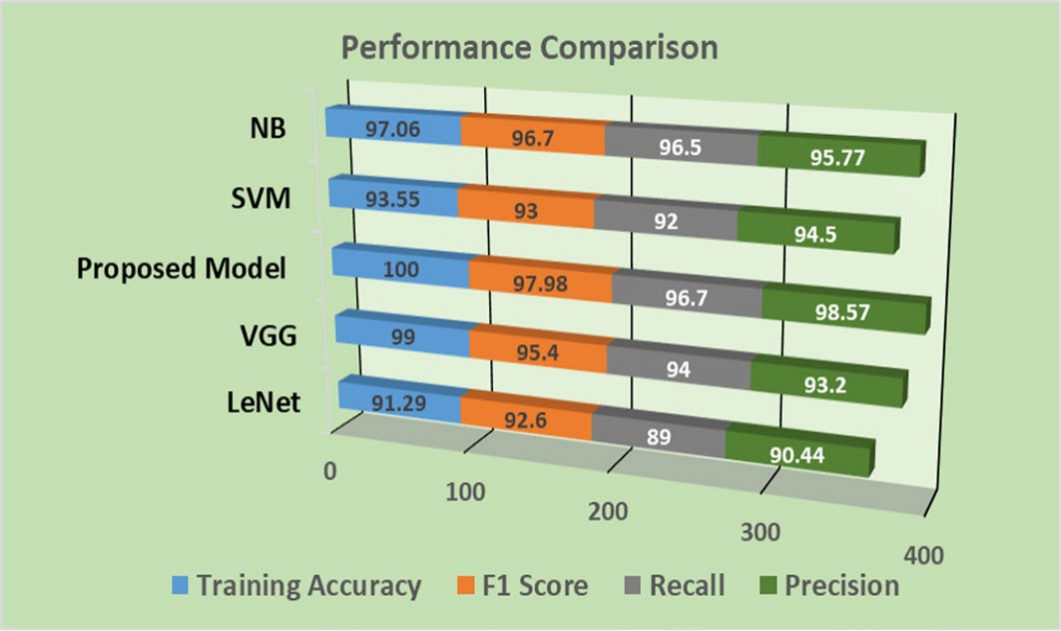
Performance evaluation of proposed algorithm in relation to others popular algorithms for cancer classification with different evaluation matrix

Figure 8 shows the mean performance comparison of all comparative and proposed model with training accuracy, F1 score, Recall and Precision. In Fig. 8 it is clearly depicted from all observation that proposed model with deep learning gives comparative good results as compared to others popular models of deep learning and machine learning for cancer classification.

**Fig. 9:**
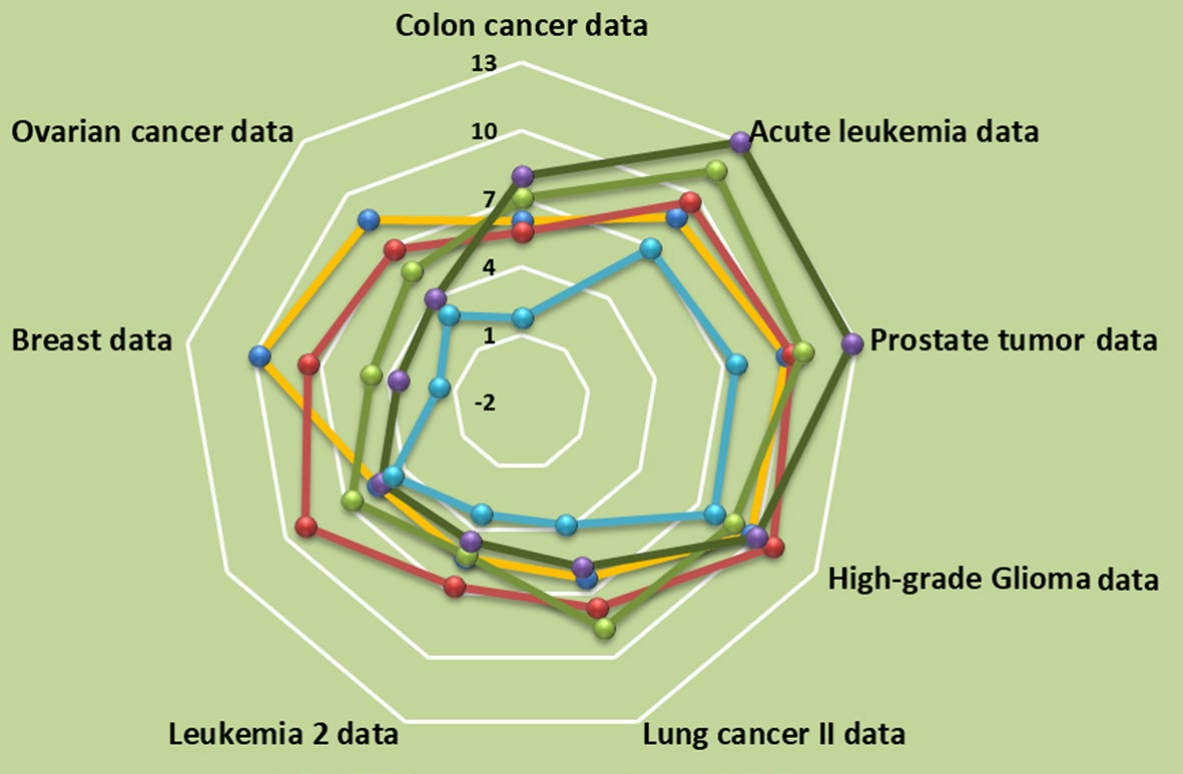
The radar plot curves of the proposed algorithm (blue line) (**e**) and the comparative classification algorithms (**a**–**d**) NB (Yellow line), SVM (Red line), VGG (Green line), LeNet (Purple line) obtained with 8 medical datasets

Figure 9 presents the radar graph that ranks the algorithms based on their error evaluation. Area near the center of the radar graph represents lower error values. Therefore, algorithms that have a narrow area that perform the best classification task, which is the proposed approach at first, followed by the VGG algorithm. The performance of the proposed approach is compared in Table 4 and the radar plot in Fig. 9, from which it can be deduced that the proposed method is superior to the established deep learning and machine learning methods.

**Table 4:** The comparison result of SVM, NB, VGG and LeNet classifiers with proposed approach

Datasets	NB	SVM	VGG	LeNet	Proposed model
	Mean classification accuracy (%)	Mean classification accuracy (%)	Mean classification accuracy (%)	Mean classification accuracy (%)	Mean classification accuracy (%)
Colon cancer data	94.12	94.11	94.01	94.21	98.27
Acute leukemia data	91.35	90.45	88.67	86.37	93.15
Prostate tumor data	90.14	89.90	89.38	87.18	92.38
High-grade Glioma data	90.32	89.22	91.24	90.04	92.16
Lung cancer II data	93.71	92.34	91.34	94.22	96.23
Leukemia 2 data	94.67	93.33	94.84	95.44	96.75
Breast data	90.23	92.44	95.22	96.43	98.32
Ovarian cancer data	91.45	93.22	94.44	96.06	96.98

### Comparison with recent published state-of-the-art feature selection algorithms

In this subsection of our experiments, we assess the performance of our proposed algorithm in comparison to state-of-the-art gene selection algorithms from the literature. The evaluation is based on classification accuracy, as detailed in Table 5. Noteworthy algorithms included for comparison are KSV-HHO [56], MRMR-MGWO [47], BGWOA [57], AD-GA [58], BCROSAT [59], Gene Vit [60], RFE-BEE [61], HAGNN [62], and ICA + CSABC [63]. Table 4 provides a comprehensive overview of the classification accuracy for our algorithm and the nine other methods across eight microarray datasets. Unknown values in the table are denoted by the character “−“. Examining Table 4 reveals that our proposed method exhibits improved accuracy compared to above including other 9 state-of-the-art gene selection algorithms. Notably, our method consistently achieves higher or equal classification accuracy across nine out of eight datasets, with exceptions observed in the Breast, and Lung cancer-II datasets. Furthermore, CSSMO achieves a theoretical optimal accuracy of 100%, accompanied by a minimum standard deviation for the aforementioned datasets.

**Table 5:** Comparison of classification accuracy of proposed algorithm with the recent published state-of-the-art feature selection algorithms

Algorithms	Colon	Lung cancer II	Acute leukemia	High-grade Glioma	Prostate	Lekuemia-2	Breast	Ovarian
CSSMO	99.36	99.07	99.28	99.82	99.98	100	98.23	96.98
kSV-HHO [51]	98.11	97.88	99.15	–	–	98.80	–	
rMRMR-MGWO [52]	95.86	97.91	–	–	–	100	–	
BGWOA [53]	100	94.97	97.7	–	–	100	80.56	94.24
AD-GA [54]	–	–	90.9	–	93.2	97.7	–	98.88
BCROSAT [55]	992.25	93.57	–	97.2	–	98.04	93.26	
GeneViT [56]	98.4	96.91	–	–	–	96.61	–	97.33
RFE-BEE [57]	99.58	99.43	–	100	–	–	–	
HAGNN [58]	98.49	98.88	–	99.05	98.85	–	91.26	93.23
ICA + CSABC [59]	99.13	93.45	98.97	97.23	100	97.63	–	

### Advantages of proposed approach

Following are the advantage of proposed algorithm:

- *Optimized gene selection:* This hybrid algorithm streamlines the identification of pertinent genes, reducing data dimensionality crucial for classification tasks. This, in turn, accelerates the process and enhances accuracy.
- *Synergistic search capabilities*: The fusion of Spider Monkey Optimization (SMO) and Cuckoo Search (CSA) amalgamates the exploration strength of SMO and the exploitation efficiency of CSA. This synergy fortifies the algorithm's robustness in locating optimal solutions.
- *Mitigated overfitting*: Through precise gene selection and noise reduction, the hybrid algorithm demonstrates reduced susceptibility to overfitting, ensuring improved generalization to unseen data.
- *Elevated model performance*: The selected genes drive a deep learning classification model, harnessing deep learning's potency for precise classification and capturing intricate data patterns.
- *Reduced computational load*: Gene selection significantly trims down the features processed by the deep learning model, resulting in expedited training and inference times.
- *Competitive accuracy*: Across diverse datasets, the hybrid approach showcases competitive or superior accuracy compared to conventional gene selection and classification methods. It excels by adeptly combining two complementary optimization techniques.
- *Versatile applications*: The algorithm's adaptability extends its utility to various classification tasks, encompassing cancer classification, disease diagnosis, and beyond, making it a valuable tool for diverse applications.

### Limitations of proposed approach

The hybrid CSSMO gene selection algorithm, designed for deep learning classification, faces several limitations. Primarily, it may encounter challenges with datasets of exceptionally high dimensionality, leading to computational and resource-intensive processes for feature selection and optimization, particularly when handling extensive multi-omics datasets. Additionally, the algorithm's performance is sensitive to parameter settings, demanding careful tuning, which may pose challenges for users lacking extensive optimization expertise. Furthermore, the interpretability of the algorithm's decisions can be complex, potentially limiting its adoption in applications prioritizing model transparency. Lastly, its efficacy may vary across diverse biological data types, lacking the exploration of biological significance discussed in references [64–66], crucial for cancer-related applications. Addressing these limitations and enhancing the algorithm's scalability, user-friendliness, and robustness are essential areas for future research and development to broaden its applications in genomics and deep learning classification.

## Conclusion

In this paper, a hybrid method for deep learning classification, named CSSMO is proposed for the utilization of feature selection. The CSSMO method is utilized in the proposed model to perform feature selection, which identifies the best subset of genes. Following that, this subset of genes is categorized by means of deep learning to identify distinct groupings or classes that are associated with a specific disease. For determining how accurate the suggested algorithm is, eight different benchmark data sets are utilized. These data sets are Colon cancer, Acute leukemia, Prostate tumor, High-grade Glioma, Lung cancer II, and Leukemia 2. We have carried out classification tests to demonstrate that the proposed model is accurate. In addition, the proposed CSSMO model’s performance was superior to that of the conventional ML and DL models that are currently being utilized. As a result, we can draw the conclusion that the proposed methodology contributes to an increase in the classification model's efficiency.

### Future research

Researchers will be able to overcome the constraints of cancer classification using gene expression data with the assistance of this method. This model has the potential to be used in the future for the purpose of enhancing accuracy by employing it as a parallel framework in conjunction with other extraction strategies in order to obtain findings that are more precise. Future research directions for the Spider Monkey and Cuckoo Search hybrid algorithm in gene selection and deep learning classification include exploring its potential for multi-omics integration, enhancing interpretability, investigating transfer learning capabilities, adapting to dynamic datasets, assessing ensemble approaches, testing real-time applications in medical diagnostics, addressing scalability, extending to cross-domain applications, leveraging hardware acceleration, considering ethical implications in medical contexts, and developing benchmark datasets for standardized performance evaluations. These avenues aim to further advance the algorithm's effectiveness, applicability, and ethical considerations in the field of genomics and deep learning-based classification. Future research will look into ways to improve accuracy by adjusting various performance metrics. Furthermore, in future work the proposed model may be evaluated on Next Generation Sequencing datasets, which can be used to sequence genomes and investigate human biomes at a much quicker and more cost-effective rate than earlier techniques.

## Data Availability

All of the aforementioned datasets are publicly available and can be accessed via the provided web links or accession numbers. We have ensured compliance with the guidelines outlined in the 'Availability of Data and Materials' section, making it convenient for readers and researchers to access the same data sources for replication and further analysis. (1) Colon Cancer: Colon Tumour Dataset. (a) Data Source: http://genomics-pubs.princeton.edu/oncology/affydata/index.html. (2) Acute Leukemia: Gene Expression Dataset (Golub et al.). (b) Data Source: https://www.kaggle.com/datasets/crawford/gene-expression. (3) Prostate Tumor: Prostate Cancer Dataset: (c) Data Source: https://ico2s.org/datasets/microarray.html. (4) High-Grade Glioma: caArray_louis-00379: Gene Expression-based Classification of Malignant Gliomas Correlates Better with Survival than Histological Classification. (d) Accession Number: GSE82009 (Gene Expression Omnibus). (5) Lung Cancer II: (e) Data Source: https://leo.ugr.es/elvira/DBCRepository/LungCancer/LungCancer-Harvard2.html. (6) Leukemia II: LEUKEMIA Dataset. (f) Data Source: https://zenodo.org/record/2709491. (7) Breast Cancer: (g) Data Source: https://www.nature.com/articles/415530a. (8) Ovarian: (h) Data Source: https://doi.org/10.1016/S0140-6736(02)07746-2.

## Abbreviations

SVM: Support vector machine
DL: Deep learning
CSA: Cuckoo search algorithm
CSSMO: Cuckoo search followed by spider monkey optimization
CNN: Convolutional neural network
SMO: Spider monkey optimization
mRMR: Minimum redundancy maximum relevance (mRMR)
GA: Genetic algorithms
PSO: Particle swarm optimization
NB: Naïve Bayes
VGG: Visual geometry group
LeNet: Simple convolutional neural network

## Code availability

All used code are freely available on net through below link (https://github.com/Rabiaaziz2023/A-Novel-and-Innovative-Cancer-Classification-Framework-with-CSSMO).
